# Psychometric properties of the Arabic version of Connor-Davidson Resilience Scale in a sample of Lebanese women

**DOI:** 10.1371/journal.pone.0269700

**Published:** 2022-06-15

**Authors:** Maya Bizri, Nour Ibrahim, Christine Beyrouthy, Dahlia Yamout, Hani Tamim, Jude Abiad, Ghina Ghazeeri

**Affiliations:** 1 Department of Psychiatry, Faculty of Medicine, American University of Beirut Medical Center, Beirut, Lebanon; 2 Department of Obstetrics and Gynecology, Faculty of Medicine, American University of Beirut Medical Center, Beirut, Lebanon; 3 Department of Internal Medicine, American University of Beirut Medical Center, Beirut, Lebanon; 4 Faculty of Sciences, Boston University, Boston, MA, United States of America; Faculty of Medicine, Saint-Joseph University, LEBANON

## Abstract

**Purpose:**

Resilience is defined as the ability to face adversities with positive outcomes. Various scales have been utilized, including 25-item Connor-Davidson Resilience Scale (CD-RISC), to evaluate resilience among populations. Resilience research is scarce, particularly in Lebanon as no such scales have been validated. Thus, in the present work, we aimed to assess the psychometric properties of the Arabic version of CD-RISC.

**Patients and methods:**

The study was conducted at the Women’s Health Center at the American University of Beirut Medical Center among Lebanese women presenting to the obstetrics and gynecology clinics. Internal validity of the Arabic CD-RISC was examined. Pearson’s correlation coefficients between the scores of the Arabic version of CD-RISC and the other related constructs (Rosenberg Self-Esteem Scale, Dispositional Hope Scale, Life Orientation Test, and Positive and Negative Affect Schedule) were assessed to evaluate its divergent and convergent validity. We collected responses from a total of 63 Lebanese women.

**Results:**

The studied scale displayed a high internal consistency. Adequate correlation coefficients were manifested by the significant positive moderate to strong and negative moderate correlations between the Arabic CD-RISC and the other related constructs.

**Conclusion:**

This is the first study to validate the Arabic version of the CD-RISC in a sample of Lebanese women. The findings of this study provide evidence that the Arabic version of CD-RISC is a reliable and valid tool for the evaluation of resilience among Lebanese women.

## Introduction

Resilience, the ability to “bounce back” from stress, as a construct has garnered increasing attention when looking at resistance to one’s risk of developing poor outcomes in the face of adversity [1]. As an adaptation process, across the lifespan, the experience of resilience will vary [2].

In the Arab World in general and in Lebanon, mental health research is focused on poor psychiatric outcomes, including rates of depression, anxiety, and post-traumatic stress syndromes. Resilience research is scarce, partly due to the lack of consensus on the scales used to measure it [2]. A recent methodological review identified three questionnaires with high ratings for their psychometric properties in capturing resilience in an adult population [2], among which is the Connor Davidson Resilience Scale (CD-RISC) [3]. No such scales have been found validated in the Arabic language. A study looking at resilience in Palestinian adolescents in Gaza [4], used ‘minimal to no anxiety and depression’ on the Arabic versions of the Beck Depression Inventory and the Beck Anxiety Inventory as a proxy to resilience.

Women’s mental health is particularly important to examine in current times. Women are more likely to develop post-traumatic stress disorder in their lifetime than men [5–8]. In light of this past year’s events in Lebanon, including the critical economic situation, due to both political turmoil and the COVID-19 pandemic, and a recently devastating atomic equivalent blast in the capital, resilience can no longer be avoided as an outcome when studying trauma in women.

Connor and Davidson (2003) developed a self-report tool to quantify resilience drawing participants from various settings. This 25-item tool is based on a five-factor structure of the resilience construct: personal competence, high standards, and tenacity; trust in one’s instincts, tolerance of negative affect, and strengthening effects of stress; positive acceptance of change and secure relationships with others; control; spiritual influences. It has been accurately translated into the Arabic language, the official language of Lebanon [9]. As such, we obtained the permission of the original authors to explore the psychometric properties of the Arabic version of the scale once applied to a Lebanese population of women. Thus, the reliability and validity of the scale in Lebanese women were assessed.

## Materials and methods

### Study design

This was a cross-sectional study conducted at the Women’s Health Center at the American University of Beirut Medical Center among Lebanese women presenting to the obstetrics and gynecology clinics between the ages of 18 and 65 between October and December 2020. We designed a questionnaire that consists of the patients’ demographics, CD-RISC scale, in addition to multiple instruments outlined before that were used for the purpose of assessing the convergent and divergent validity of CD-RISC. Ethical approval was granted by the Institutional Review Board (IRB) of the Human Research Protection Program (HRPP) at the American University of Beirut (IRB Number: SBS-2020-0407).

### Instruments

#### Resilience

As aforementioned, the *Connor-Davidson Resilience Scale (CD-RISC)* Arabic version was used. It was obtained with permission from the original authors. The scale consists of 25 items included in the original form. Items are rated from 0–4 on a 5-point Likert scale. Higher scores indicate higher resilience. The original scale showed a calculated Cronbach alpha of 0.89 in the general population.

#### Global self-worth

One of the main constructs defining psychological wellbeing is self-esteem. The *Rosenberg Self-Esteem Scale (RSES)*, measures the subjective evaluation of one’s self-worth [10, 11]. The scale contains 10 items, all items are on a 4-point Likert scale, and higher scores are correlated with better self-worth. RSES has been shown to have good validity and reliability among different samples including Arabic-speaking populations [12, 13]. We obtained permission to use the back translated Arabic version from authors of Zayed et al, 2016. Since self-esteem is usually perceived as an indicator of better mental health, resilience was found to be positively correlated with self-esteem [14]. The Pearson’s correlation coefficient between self-esteem and resilience using the Rosenberg scale was 0.53 (N = 246, p<0.001) [15].

#### Dispositional hope

Hopefulness is at the core of the ability to recover after adversity. It was assessed using the Arabic version of the *Dispositional Hope Scale (DHS)* [16]. The 12-item self-report scale has been widely used in various populations including Arabic speaking populations [17]. The Arabic version was validated in a Lebanese population with an internal consistency of alpha = 0.87 [18]. Permission to use the latter was obtained from authors of Ilyas & Kazarian, 2017. Hope is positively correlated with resilience as observed using the Dispositional Hope Scale (N = 246, Pearson r = 0.68, p<0.001) [15].

#### Optimism

Optimism, the cognitive outlook at the core of favorable outcomes, is also at the core of resilience. *Life Orientation Test (LOT)* [19] and its revised form *(LOT-R)* are the most widely used measures of optimism. The 10-item self-reported Arabic LOT-R consists of three optimism items, three pessimism items, and four filler items. Respondents rate items on a 5-point Likert scale, with higher scores indicating a greater disposition for a positive outlook. We obtained permission from Ilyas & Kazarian (2017) to use the Arabic version, which had an internal consistency of alpha = 0.62. The English version had a reported internal consistency of 0.78 [20]. The Life Orientation scale was found to have positive correlation with resilience as optimism is linked to resilience in general (N = 246, Pearson r = 0.55, p<0.001) [15].

#### Positive and Negative Affect

The *Positive and Negative Affect Schedule (PANAS)* [21] is a 20-item scale consisting of two subscales, each having 10 affective descriptors. Positive Affect indicates emotional wellbeing, including joy, inspiration, enthusiasm, whereas the Negative Affect subscale measures emotional distress, including anger, guilt, disgust and anxiety. Each item is rated on a 5-point Likert scale, and higher scores indicate a higher subscale measure. We obtained permission to use the Arabic version of the PANAS from Ilyas & Kazarian (2017). Comparing resilience with positive and negative emotions showed that CD-RISC was positively correlated with a positive affect score (N = 246, Pearson r = 0.69, p<0.001), and negatively correlated with a negative affect score (N = 246, Pearson r = 0.44, p<0.001) [15].

### Statistical analysis plan

Statistical analysis was performed using SPSS 25 statistical software package (IBM, USA). Descriptive analyses were performed for all scores obtained from the Arabic version of CD-RISC. Pearson’s correlation coefficients between the scores of the Arabic version of CD-RISC and other related constructs were calculated for the evaluation of divergent and convergent validity. Also, inter-item correlations and internal validity were examined. Level of significance was set at α = 0.05.

## Results

**Fig 1:**
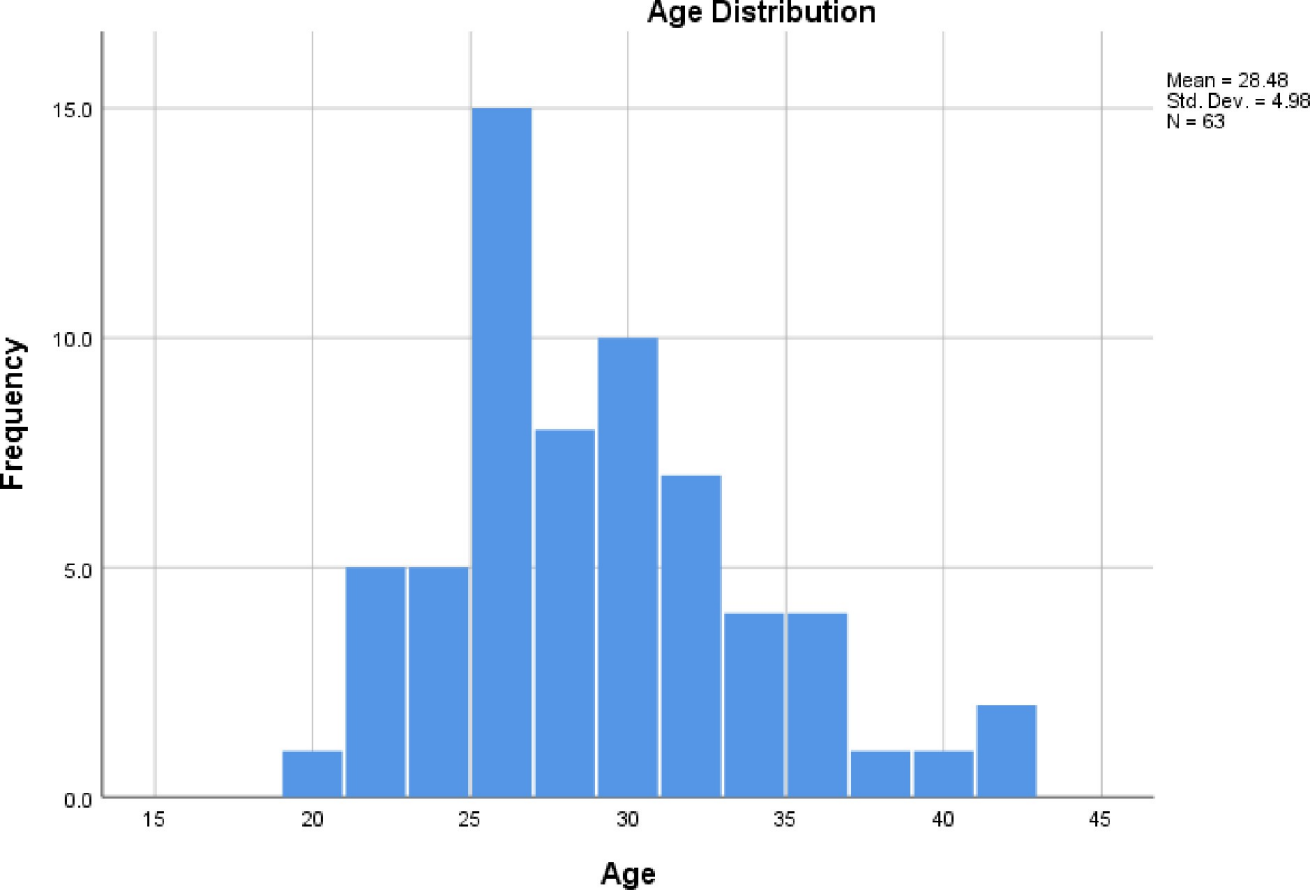
The age distribution of our sample population.

A total of 63 Lebanese women participated in this CD-RISC validation study. Participants had a mean age of 28.48 ± 4.98 years. The age distribution of the sample population is presented in **Fig 1**. **Table 1**summarizes the scores of the studied scales among our sample population.

**Table 1:** Descriptive analyses of the studied scales among a sample of Lebanese women.

	Mean ± Standard Deviation	Range
CD-RISC score, [0–100]	69.63 ± 12.26	36–97
RSES score, [0–30]	23.74 ± 4.51	10–30
LOT score, [0–24]	14.59 ± 3.76	2–24
DHS score, [0–100]	67.98 ± 6.86	49–87
PANAS–Positive score, [0–50]	36.01 ± 7.33	14–50
PANAS–Negative score, [0–50]	26.25 ± 7.65	10–49

CD-RISC: Connor-Davidson Resilience Scale; RSES: Rosenberg Self-Esteem Scale; LOT: Life Orientation Test; DHS: Dispositional Hope Scale; PANAS: Positive and Negative Affect Schedule.

### Reliability

The Cronbach’s alpha for the Arabic version of CD-RISC Arabic was reported to be 0.89 (95% confidence interval [CI]: 0.832–0.919). Results showed that the 25 items of the Arabic CD-RISC had a relatively high internal consistency among our participants.

### Convergent validity

We present the Pearson correlation coefficients (r) for Arabic CD-RISC and the following scales measuring self-esteem (RSES), dispositional hope (DHS), optimism (LOT), and positive affect (PANAS) in **Table 2**.

**Table 2:** The Pearson correlation coefficients between Arabic CD-RISC with the other studied scales.

		CD-RISC	RSES	LOT	DHS	PANAS–Positive Affect	PANAS–Negative Affect
CD-RISC	r	1	0.559	0.536	0.494	0.633	-0.390
	p-value		**<0.0001**	**<0.0001**	**<0.0001**	**<0.0001**	**<0.0001**
RSES	r		1	0.566	0.372	0.514	-0.451
	p-value			**<0.0001**	**<0.0001**	**<0.0001**	**<0.0001**
LOT	r			1	0.328	0.396	-0.445
	p-value				**<0.0001**	**<0.0001**	**<0.0001**
DHS	r				1	0.417	-0.030
	p-value					**<0.0001**	0.634
PANAS–Positive Affect	r					1	-0.210
	p-value						**0.001**
PANAS–Negative Affect	r						1

r = Pearson Correlation; CD-RISC: Connor-Davidson Resilience Scale; RSES: Rosenberg Self-Esteem Scale; LOT: Life Orientation Test; DHS: Dispositional Hope Scale; PANAS: Positive and Negative Affect Schedule.

The highest correlation was found between CD-RISC and positive affect of PANAS (r = 0.63, p <0.0001). Similarly, each of the scales RSES, LOT, DHS revealed a moderately strong correlation with CD-RISC (r = 0.56, 0.54 and 0.5 respectively).

### Divergent validity

CD-RISC possessed a significant yet weak-moderate negative correlation with the negative affect items of PANAS (r = -0.4, p <0.0001).

## Discussion

The objective of this study was to evaluate the psychometric properties of the Arabic version of the CD-RISC in a Lebanese sample of women. The results indicated that the reliability and validity of the Arabic version of the CD-RISC are adequate among our studied Lebanese women sample.

Establishing the internal consistency of the Arabic version in a Lebanese population is essential for future evaluation of resilience. The reliability of the used Arabic CD-RISC is comparable to the results of the original research that developed the adapted Arabic version of the CD-RISC resilience instrument [9]. In fact, Cronbach’s alpha is almost identical in both studies (0.89).

Convergent and discriminant validities of the used scale were computed to determine its validity among the studied sample. The convergent validity of Arabic CD-RISC was confirmed by the significant positive correlation with the positive affect of the PANAS scale. This comes in line with several studies revealing that highly resilient people cultivate their positive emotionality to overcome adverse events [22]. This makes positive affect an essential unit of the psychological resilience of an individual. Although moderate, the negative correlation between the negative affect of PANAS and CD-RISC establishes an extension of the findings where resilience is not considered a trait only but also a process based on myriad stressors and the negative affects coupled with it [23]. Similarly, a moderate correlation coefficient existed between CD-RISC and each of RSES, DHS, and LOT. Indeed, the latter are closely aligned constructs with resilience, as they all correlate with positive emotionality and optimistic outlook against hurdles [24–26].

The validation of the CD-RISC in the Arabic language has great implications on future resilience research in Lebanon and the region. Since its inception, Lebanon has been crippled by constant internal and external conflicts and instabilities. It has been repeatedly shaken by armed conflicts, unrest, and turmoil over the decade, leaving a notable mark on its people at both physical and psychological levels [27]. Nevertheless, the Lebanese people have never before experienced a more dreadful event than the recent August 4th, 2020 Beirut explosion [27]. The explosion was preceded by the October 17th, 2019 revolution and followed by an economic crisis, a global pandemic, and political violence [27].

However, despite the extensive history of economic and political grievances, studies conducted over the past 50 years have shown that Lebanese people tend to adapt fairly well to hardships [28]. This has been eminent in their ability to maintain functionality in academic, social, and occupational settings. It has been proposed that the Lebanese people’s ability to cope with distress has been part of the Lebanese identity transmitted through generations [29]. This might provide them with a sense of coherence and hope in their future. However, there hasn’t been any empirical studies that examined these claims on the Lebanese population, as these explanations were theorized by authors but were not backed up by neither quantitative nor qualitative data.

Literature suggests that childhood and adolescence are two critical developmental periods of heightened brain plasticity and increased sensitivity to environmental stressors [30, 31]. Exposure to adversities during these two developmental periods affect the stress response system and brain circuit that mediates emotional regulation [30, 32]. Studies in children who show features of resilience despite early trauma have identified crucial psychosocial factors that promote resilience, most prominently the formation of a solid bond with parents and family stability [33, 34]. The Lebanese culture is known to be collectivistic, with family support and values playing a salient role in the child’s life. This culture might be a reason for the resilient nature of the Lebanese people as they rely highly on psychosocial factors to promote coping with hardships. As evident from the above, the validated Arabic version of the CD-RISC is essential to further examine these claims on the Lebanese population after the cascade of devastating events that occurred over the past 2 years. Having an adequate instrument to measure resilience in the Lebanese population will prove to further advance empirical research examining the features and drivers of this apparent resilience.

Further, it is important to point out some limitations. The data was collected from a single center and the majority of our participants had high educational level and good socioeconomic status. Also, the sample size was limited. This may decrease the generalizability of the results.

## Conclusion

This is the first study to validate the Arabic version of the Connor-Davidson Resilience Scale. We propose that the Arabic version of the CD-RISC has good psychometric properties. Although the study presents limitations, the Arabic version of the CD-RISC is a reliable and valid tool to evaluate resilience in Arabic-speaking women. Future studies should focus on empirically evaluating resilience, sense of coherence, and hope in the Lebanese population after the events of the last two years and consequently investigating the implications such events had on mental health. In addition, examining protective and moderating factors such as coping mechanisms is essential to fill in the gap in the trauma literature in the region.

## References

[1] Charney DS. Psychobiological Mechanisms of Resilience and Vulnerability: Implications for Successful Adaptation to Extreme Stress. Am J Psychiatry [Internet]. 2004 Feb;161(2):195–216. Available from http://psychiatryonline.org/doi/abs/10.1176/appi.ajp.161.2.195 14754765 10.1176/appi.ajp.161.2.195

[2] Windle G, Bennett KM, Noyes J. A methodological review of resilience measurement scales. Health Qual Life Outcomes [Internet]. 2011;9(1):8. Available from http://hqlo.biomedcentral.com/articles/10.1186/1477-7525-9-8 21294858 10.1186/1477-7525-9-8 PMC3042897

[3] Connor KM, Davidson JRT. Development of a new resilience scale: The Connor-Davidson Resilience Scale (CD-RISC). Depress Anxiety [Internet]. 2003 Sep;18(2):76–82. Available from https://onlinelibrary.wiley.com/doi/10.1002/da.10113 12964174 10.1002/da.10113

[4] Aitcheson RJ, Abu-Bader SH, Howell MK, Khalil D, Elbedour S. Resilience in Palestinian adolescents living in Gaza. Psychol Trauma Theory, Res Pract Policy [Internet]. 2017 Jan;9(1):36–43. Available from http://doi.apa.org/getdoi.cfm?doi=10.1037/tra0000153 27243568 10.1037/tra0000153

[5] Christiansen DM, Hansen M. Accounting for sex differences in PTSD: A multi-variable mediation model. Eur J Psychotraumatol [Internet]. 2015 Dec 19;6(1):26068. Available from https://www.tandfonline.com/doi/full/10.3402/ejpt.v6.26068 25604705 10.3402/ejpt.v6.26068 PMC4300366

[6] Ditlevsen DN, Elklit A. The combined effect of gender and age on post traumatic stress disorder: do men and women show differences in the lifespan distribution of the disorder? Ann Gen Psychiatry [Internet]. 2010 Dec 21;9(1):32. Available from https://annals-general-psychiatry.biomedcentral.com/articles/10.1186/1744-859X-9-32 20663164 10.1186/1744-859X-9-32 PMC2917414

[7] Olff M, Langeland W, Draijer N, Gersons BPR. Gender differences in posttraumatic stress disorder. Psychol Bull [Internet]. 2007;133(2):183–204. Available from http://doi.apa.org/getdoi.cfm?doi=10.1037/0033-2909.133.2.183 17338596 10.1037/0033-2909.133.2.183

[8] Tolin DF, Foa EB. Sex differences in trauma and posttraumatic stress disorder: A quantitative review of 25 years of research. Psychol Bull [Internet]. 2006;132(6):959–92. Available from http://doi.apa.org/getdoi.cfm?doi=10.1037/0033-2909.132.6.959 17073529 10.1037/0033-2909.132.6.959

[9] Toma G, Guetterman TC, Yaqub T, Talaat N, Fetters MD. A systematic approach for accurate translation of instruments: Experience with translating the Connor–Davidson Resilience Scale into Arabic. Methodol Innov [Internet]. 2017 Jul 21;10(3):205979911774140. Available from http://journals.sagepub.com/doi/10.1177/2059799117741406

[10] Rosenberg M. Society and the Adolescent Self-Image. Princeton, New Jersey: Princeton University Press; 1965.

[11] Rosenberg M. Society and the Adolescent Self-Image. Revised Ed. Middletown, CT: Wesleyan University Press; 1989.

[12] Al-Fayez GA, Ohaeri JU, Gado OM. Prevalence of physical, psychological, and sexual abuse among a nationwide sample of Arab high school students: association with family characteristics, anxiety, depression, self-esteem, and quality of life. Soc Psychiatry Psychiatr Epidemiol [Internet]. 2012 Jan 13;47(1):53–66. Available from http://link.springer.com/10.1007/s00127-010-0311-2 21076913 10.1007/s00127-010-0311-2

[13] Zayed KN, Al-Busafi M, Al Haddabi B, Al-Rawahi N, Al-Tauqi M, Thiyabat F. Gender Differences in Self-Esteem and its Relationship with Body Mass Index among Omani Adolescents. Can J Clin Nutr [Internet]. 2016 Jan;4(1):18–24. Available from http://globalscienceheritage.org/downloads/gender-differences-in-self-esteem-and-its-relationship-with-body-mass-index-among-omani-adolescents/ doi: 10.14206/canad.j.clin.nutr.2016.01.03

[14] Benetti C, Kambouropoulos N. Affect-regulated indirect effects of trait anxiety and trait resilience on self-esteem. Pers Individ Dif [Internet]. 2006 Jul;41(2):341–52. Available from https://linkinghub.elsevier.com/retrieve/pii/S0191886906000857 doi: 10.1016/j.paid.2006.01.015

[15] Karaırmak Ö. Establishing the psychometric qualities of the Connor–Davidson Resilience Scale (CD-RISC) using exploratory and confirmatory factor analysis in a trauma survivor sample. Psychiatry Res [Internet]. 2010 Oct;179(3):350–6. Available from https://linkinghub.elsevier.com/retrieve/pii/S0165178109003576 doi: 10.1016/j.psychres.2009.09.012 20493533

[16] Snyder CR, Harris C, Anderson JR, Holleran SA, Irving LM, Sigmon ST, et al. The will and the ways: Development and validation of an individual-differences measure of hope. J Pers Soc Psychol [Internet]. 1991;60(4):570–85. Available from http://doi.apa.org/getdoi.cfm?doi=10.1037/0022-3514.60.4.570 2037968 10.1037//0022-3514.60.4.570

[17] Abdel-Khalek A, Snyder† CR. Correlates and predictors of an Arabic translation of the Snyder Hope Scale. J Posit Psychol [Internet]. 2007 Oct;2(4):228–35. Available from http://www.tandfonline.com/doi/abs/10.1080/17439760701552337

[18] Ilyas RR, Kazarian SS. A validation study of a new Arabic version of the adult dispositional hope scale in a sample of Lebanese college youth. Arab J Psychiatry [Internet]. 2017;28(1):83–91. Available from https://search.emarefa.net/detail/BIM-795138

[19] Scheier MF, Carver CS. Optimism, coping, and health: Assessment and implications of generalized outcome expectancies. Heal Psychol [Internet]. 1985;4(3):219–47. Available from http://doi.apa.org/getdoi.cfm?doi=10.1037/0278-6133.4.3.219 10.1037//0278-6133.4.3.219 4029106

[20] Scheier MF, Carver CS, Bridges MW. Distinguishing optimism from neuroticism (and trait anxiety, self-mastery, and self-esteem): A reevaluation of the Life Orientation Test. J Pers Soc Psychol [Internet]. 1994;67(6):1063–78. Available from http://doi.apa.org/getdoi.cfm?doi=10.1037/0022-3514.67.6.1063 7815302 10.1037//0022-3514.67.6.1063

[21] Watson D, Clark LA, Tellegen A. Development and validation of brief measures of positive and negative affect: The PANAS scales. J Pers Soc Psychol [Internet]. 1988;54(6):1063–70. Available from http://doi.apa.org/getdoi.cfm?doi=10.1037/0022-3514.54.6.1063 3397865 10.1037//0022-3514.54.6.1063

[22] Gloria CT, Steinhardt MA. Relationships among Positive Emotions, Coping, Resilience and Mental Health. Stress Heal. 2016;32(2):145–56. doi: 10.1002/smi.2589 24962138

[23] Chen D, Wu J, Yao Z, Lei K, Luo Y, Li Z. Negative association between resilience and event-related potentials evoked by negative emotion. 2018;(November 2017):1–6. 10.1038/s41598-018-25555-w PMC5940768 29740037

[24] Youssef CM, Luthans F. Positive organizational behavior in the workplace: The impact of hope, optimism, and resilience. J Manage. 2007;33(5):774–800.

[25] Coutu DL. How resilience works. Harv Bus Rev [Internet]. 2002 May;80(5):46–50, 52, 55 passim. Available from http://www.ncbi.nlm.nih.gov/pubmed/12024758 12024758

[26] Khampirat B. The relationship between paternal education, self-esteem, resilience, future orientation, and career aspirations. PLoS One [Internet]. 2020;15(12):e0243283. Available from http://www.ncbi.nlm.nih.gov/pubmed/33290431 doi: 10.1371/journal.pone.0243283 33290431 PMC7723283

[27] Farran N. Mental health in Lebanon: Tomorrow’s silent epidemic. Ment Heal Prev [Internet]. 2021 Dec;24:200218. Available from https://linkinghub.elsevier.com/retrieve/pii/S2212657021000222 doi: 10.1016/j.mhp.2021.200218 34660191 PMC8503814

[28] El Sayed NM, Puffer ES, Sikkema KJ. The ecology of resilience: Predictors of psychological health in youth in Lebanon. Community Psychol Glob Perspect. 2018;4(2):136–49.

[29] Doumit R, Afifi RA, Devon HA. Serenity in Political Uncertainty. Holist Nurs Pract [Internet]. 2015 Mar;29(2):78–86. Available from https://journals.lww.com/00004650-201503000-00004 doi: 10.1097/HNP.0000000000000077 25658930

[30] Gee DG, Casey BJ. The impact of developmental timing for stress and recovery. Neurobiol Stress [Internet]. 2015 Jan;1:184–94. Available from https://linkinghub.elsevier.com/retrieve/pii/S2352289515000235 doi: 10.1016/j.ynstr.2015.02.001 25798454 PMC4363736

[31] Tottenham N. The importance of early experiences for neuro-affective development. Curr Top Behav Neurosci [Internet]. 2014;16:109–29. Available from http://link.springer.com/10.1007/7854_2013_254 24264369 10.1007/7854_2013_254 PMC4021037

[32] van Bodegom M, Homberg JR, Henckens MJAG. Modulation of the Hypothalamic-Pituitary-Adrenal Axis by Early Life Stress Exposure. Front Cell Neurosci [Internet]. 2017 Apr 19;11. Available from http://journal.frontiersin.org/article/10.3389/fncel.2017.00087/full 28469557 10.3389/fncel.2017.00087 PMC5395581

[33] Sapienza JK, Masten AS. Understanding and promoting resilience in children and youth. Curr Opin Psychiatry [Internet]. 2011 Jul;24(4):267–73. Available from http://journals.lww.com/00001504-201107000-00003 doi: 10.1097/YCO.0b013e32834776a8 21546838

[34] Lind MJ, Brown RC, Sheerin CM, York TP, Myers JM, Kendler KS, et al. Does Parenting Influence the Enduring Impact of Severe Childhood Sexual Abuse on Psychiatric Resilience in Adulthood? Child Psychiatry Hum Dev [Internet]. 2018 Feb 9;49(1):33–41. Available from http://link.springer.com/10.1007/s10578-017-0727-y 28488144 10.1007/s10578-017-0727-y PMC5680128

